# Predictors of Health-Related Quality of Life After Coronary Artery Bypass Grafting: A Systematic Review

**DOI:** 10.3390/jcm15218628

**Published:** 2026-11-05

**Authors:** Dimitrios Argyropoulos, Arian Arjomandi Rad, Antonios Kourliouros, Dionysios Pavlopoulos, Ioannis Toumpoulis, Dimitrios Angouras

**Affiliations:** 1 Department of Cardiac Surgery, Attikon Hospital, National and Kapodistrian University of Athens, 115 27 Athens, Greece; argiropoulosdimi@gmail.com (D.A.); a.kourliouros@gmail.com (A.K.); dpavlopoulos@yahoo.gr (D.P.); toumpoul@otenet.gr (I.T.); dangouras@yahoo.com (D.A.); 2 Department of Cardiothoracic Surgery, John Radcliffe Hospital, Oxford University Hospitals NHS Foundation Trust, Oxford OX3 9DU, UK

**Keywords:** coronary artery bypass grafting, quality of life, SF-36, cardiac surgery, systematic review, PRISMA, patient-reported outcome measures

## Abstract

**Background**: Coronary artery bypass grafting (CABG) is the most common adult cardiac surgical procedure worldwide. Although survival and graft patency are well characterised, health-related quality of life (HRQoL) is increasingly recognised as a patient-centred outcome that may be modifiable. Existing syntheses have summarised global HRQoL change after CABG but have not consistently focused on the patient-, surgery- and recovery-level factors associated with that change. **Methods**: PubMed/MEDLINE, Embase, Cochrane CENTRAL and CINAHL were searched from 1 January 2010 to 31 March 2025. Eligible studies were primary investigations (prospective or retrospective cohort, cross-sectional analytical, or randomised controlled trial) of adults (≥18 years) undergoing isolated CABG that reported HRQoL with a validated multidimensional instrument and assessed at least one candidate factor. Two reviewers independently screened records, extracted data and assessed risk of bias using the Newcastle–Ottawa Scale for observational studies and the Cochrane Risk of Bias 2 tool for randomised trials. Certainty of evidence was rated using the GRADE approach as adapted for prognostic-factor questions. Owing to heterogeneity in instruments, factor definitions and follow-up, results were synthesised narratively; no meta-analysis was performed. **Results**: Of 1220 records identified, six studies met the inclusion criteria, three prospective cohorts, two cross-sectional analytical studies and one randomised controlled trial, together contributing analysable HRQoL data on 1271 patients. Follow-up ranged from the end of post-operative cardiac rehabilitation to 10 years. Four different instruments were used: the SF-36 in three studies, the SF-36 with the SF-12 in one, the SF-12v2 in one and the disease-specific MacNew questionnaire in one. Risk of bias was low in two cohort studies and moderate in the remaining three observational studies, and low overall for the randomised trial. No factor was examined by more than five of the six studies, and most were reported by only one or two; findings are therefore described by the number of supporting studies rather than as consistent. Diabetes mellitus and post-operative complications were each independently associated with poorer physical HRQoL in two studies. Female sex, older age, lower pre-operative HRQoL, repeat percutaneous coronary intervention, heart failure or dyspnoea, social support, cardiac self-efficacy, marital status and unemployment were each supported by a single study. Depressive symptoms were not confirmed as an independent factor in the only study modelling them separately, and cardiac rehabilitation could not be evaluated because every patient received it wherever it was delivered. The sole randomised comparison showed that exercise-based prehabilitation improved pre-operative functional capacity and quality of life, but by less than the minimal clinically important difference and with no significant post-operative effect. **Conclusions**: Factors associated with HRQoL after CABG cluster into non-modifiable demographic and comorbidity factors, potentially modifiable psychosocial and behavioural factors, and recovery-related factors emerging post-operatively. The evidence base is nevertheless small, clinically and methodologically heterogeneous, and almost entirely observational. The factors identified are candidate targets requiring confirmation in prospective studies with pre-operative measurement and, thereafter, in randomised trials; they are not established causal determinants. Persistent heterogeneity of HRQoL instruments and factor definitions argues for standardised reporting in future studies of cardiac surgical recovery.

## 1. Introduction

Cardiovascular disease remains the leading cause of mortality worldwide, and ischaemic heart disease accounts for a substantial and growing share of that burden [1,2,3]. Coronary artery bypass grafting (CABG) is the most frequently performed adult cardiac surgical procedure, and its survival advantage over percutaneous coronary intervention in patients with multivessel disease—particularly those with diabetes and higher anatomical complexity—is well established [4].

Health-related quality of life (HRQoL) has emerged as a key patient-centred outcome. HRQoL captures the multidimensional impact of disease and its treatment on physical functioning, symptom burden, psychological well-being and social participation, and is most commonly quantified in cardiac surgery using the 36-item Short-Form Health Survey (SF-36) [5]. Because survival after elective CABG is now high, the marginal value of the operation to an individual patient is increasingly expressed in terms of functional recovery rather than mortality alone. Prolonged intensive care stay and post-operative complications are recognised determinants of adverse outcome after cardiac surgery [6], and early work using the SF-36 demonstrated that pre-operative health status, comorbidity and social circumstances all shape recovery at one year [7].

Two previous syntheses have informed this question. Schmidt-RioValle and colleagues reviewed 16 studies and meta-analysed three to describe global HRQoL change after CABG, demonstrating clinically meaningful average improvement but without focusing on patient-level predictors [8]. Sanders and colleagues systematically reviewed 41 studies across the broader spectrum of adult cardiac surgery and identified 103 independent predictors, but the inclusion of valve and combined procedures introduced clinical heterogeneity [9]. Neither synthesis was restricted to isolated CABG.

Furthermore, the field has produced a number of methodologically improved primary studies since 2020, including cohorts that stratify analyses by isolated CABG and randomised evidence for pre-operative exercise interventions. A focused synthesis restricted to isolated CABG, applying a single risk-of-bias framework and reported in accordance with the PRISMA 2020 statement [10], is therefore warranted.

### Objectives

The objective of this systematic review was to identify, appraise and synthesise primary studies reporting on factors associated with HRQoL in adults undergoing isolated CABG, and to distinguish factors that are potentially modifiable from those that are not. A further objective was to classify each factor by the type of evidence supporting it—cross-sectional association, prognostic association, or randomised intervention effect—so that correlates are not mistaken for demonstrated therapeutic targets.

## 2. Materials and Methods

### 2.1. Protocol and Reporting

This systematic review was conducted and reported in accordance with the Preferred Reporting Items for Systematic Reviews and Meta-Analyses (PRISMA) 2020 statement [10] (Table S1). Eligibility criteria, search strategy, outcomes and the planned approach to synthesis were agreed by all authors before the search was executed. The review was not prospectively registered in a public registry.

### 2.2. Terminology: Association, Prognostic Factor and Intervention Effect

Throughout this review, we distinguish three concepts that are frequently conflated in the quality-of-life literature, following the framework set out by the PROGRESS group [11]. An association is a statistical relationship between a variable and HRQoL observed within a study population, with no claim about temporal ordering or causation; a cross-sectional design can establish only this. A prognostic association is one in which the candidate variable is measured before the outcome, so that it can be used to stratify patients by expected outcome; prospective cohorts with pre-operative measurement support this stronger interpretation. An intervention effect is the change in HRQoL produced by deliberately manipulating a factor, and can be established only by randomised allocation or by designs with strong control of confounding.

We applied these labels to every factor identified and reserve the word predictor for variables measured before the outcome. Factors identified only in cross-sectional analyses are described as associated with HRQoL rather than as predictors of it. Because only one included study was randomised, only one factor in this review—exercise-based prehabilitation—rests on evidence capable of establishing an intervention effect. This distinction is carried through the Section 3, the certainty ratings and the Section 5.

### 2.3. Eligibility Criteria

Eligibility was framed using the PICOS approach (Table 1).

The 1 January 2010 lower bound was selected to capture studies reporting on contemporary surgical, peri-operative care and rehabilitation practices, which differ materially from those of earlier eras.

### 2.4. Information Sources

Four electronic bibliographic databases were searched from 1 January 2010 to 31 March 2025: PubMed/MEDLINE, Embase (Elsevier), the Cochrane Central Register of Controlled Trials (CENTRAL) and CINAHL (EBSCO). Reference lists of the two previous relevant systematic reviews [8,9] were hand-searched, and forward citation searching was performed on all included studies.

### 2.5. Search Strategy

Search terms combined three concepts using Boolean operators: (i) coronary artery bypass grafting, (ii) quality of life, and (iii) predictors or determinants. Database-specific strategies are reported in full in Table 2.

### 2.6. Selection Process

All identified records were imported into Rayyan (Rayyan Systems Inc., Cambridge, MA, USA) for de-duplication and screening. Two reviewers independently screened titles and abstracts, and then full texts, against the eligibility criteria. Disagreements were resolved by discussion and, where necessary, by a third reviewer.

### 2.7. Data Extraction

A standardised data extraction form was piloted on two included studies and refined before full extraction. Two reviewers independently extracted study characteristics, population details, HRQoL instrument and timing, factors examined, effect estimates and risk-of-bias information. Discrepancies were resolved by consensus. Data were extracted from the full text and Supplementary Materials of each report, and where a study reported an analysis for both a whole cohort and an isolated-CABG subgroup, the subgroup was used to define the population contributed to this review.

### 2.8. Risk-of-Bias Assessment

Risk of bias was assessed independently by two reviewers. For observational studies (prospective cohort, retrospective cohort, cross-sectional), the Newcastle–Ottawa Scale (NOS) was used, applying the cohort version and the cross-sectional adaptation as appropriate [12]. The randomised controlled trial was assessed with the Cochrane Risk of Bias 2 (RoB 2) tool.

### 2.9. Outcome Measures and HRQoL Instruments

The primary outcome was HRQoL, measured either as a global or component score (for example, the SF-36 physical component summary [PCS] and mental component summary [MCS]) or at the level of individual domains. SF-36 scores were interpreted using established norm-based scoring conventions [13]. Because the included studies used four different instruments, scores are reported and interpreted within each instrument and are not converted between them.

### 2.10. Data Synthesis

We pre-specified that quantitative synthesis (meta-analysis) would be conducted if at least three studies reported the same factor measured on a comparable scale with a comparable outcome metric. This threshold was not met for any factor. Results were therefore synthesised narratively and reported in accordance with the Synthesis Without Meta-analysis (SWiM) guideline [14]. Because no meta-analysis was performed, formal assessment of small-study effects by funnel plot was not applicable [15].

In addition to the planned synthesis, clinical and methodological heterogeneity was assessed explicitly across five pre-specified domains—study design, population and case-mix, HRQoL instrument, timing of outcome assessment, and factor definition and statistical modelling. This assessment is reported in Section 3.3. For every factor we also recorded the number of studies that examined it and, separately, the number that reported an independent association, so that a factor tested and not confirmed can be distinguished from one never measured.

### 2.11. Certainty of Evidence

Certainty of evidence for each factor was rated using the GRADE approach as adapted for questions of prognosis and prognostic factors [16,17,18]. We adopted this adaptation rather than the intervention version of GRADE because the review question concerns the association between patient- and care-level factors and a subsequent patient-reported outcome, not the effect of an intervention. Three features of the adaptation are material to our ratings and are stated explicitly here.

First is starting certainty. Under GRADE Guidelines 28, a body of observational evidence addressing a prognostic-factor question begins at high certainty rather than low, because for such questions a well-conducted cohort study is the appropriate rather than a second-best design [18]. We therefore started each factor at high certainty and rated down. This differs from the intervention framework and explains why observational findings here can retain moderate certainty.

Second is the rating-down domains. We applied the five standard domains: risk of bias (assessed with the tools in Section 2.8); inconsistency in the direction or magnitude of association across studies; indirectness of population, factor or outcome relative to our review question, under which we rated down factors derived solely from cross-sectional designs because the temporal ordering required for a prognostic claim is absent; imprecision of the reported estimates; and publication or selective-reporting bias. Following Huguet and colleagues [17], we additionally considered phase of investigation, rating down where a factor was examined only in exploratory analyses or entered through stepwise selection rather than in a model specified a priori.

Third is the rating-up domains. We considered a large magnitude of effect and an exposure–response gradient but did not rate up on either ground for any factor, because no factor was supported by estimates of sufficient size and precision across independent studies to justify it.

Two reviewers rated each factor independently and disagreements were resolved by discussion. We acknowledge three limitations of this approach. GRADE for prognostic factors is less thoroughly validated than its intervention counterpart; judgements about indirectness and phase of investigation are necessarily subjective; and with only six studies the inconsistency domain is poorly informed. Certainty ratings should therefore be read as a structured and transparent summary of our confidence rather than as a precise metric.

## 3. Results

### 3.1. Study Selection

**Figure 1:**
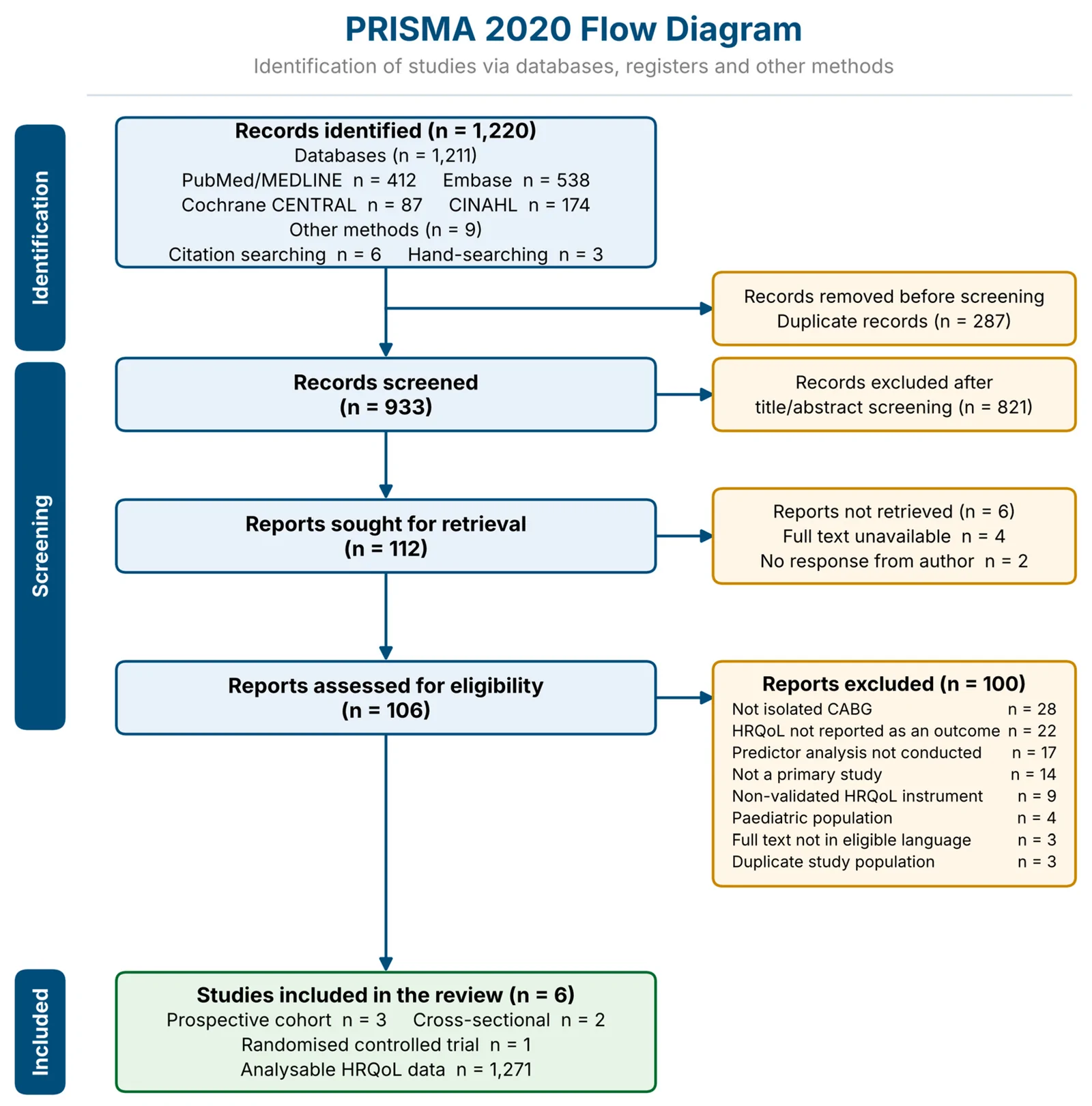
PRISMA 2020 flow diagram showing the identification, screening, eligibility assessment and inclusion of studies. The final box reports the number of patients contributing analysable HRQoL data (n = 1271).

The database search yielded 1211 records (PubMed/MEDLINE n = 412; Embase n = 538; Cochrane CENTRAL n = 87; CINAHL n = 174), with an additional nine records identified from citation searching (n = 6) and hand-searching of reference lists of previous reviews (n = 3), giving 1220 records in total. After removal of 287 duplicates, 933 unique records entered title and abstract screening, of which 821 were excluded. Of the 112 reports sought for retrieval, six could not be obtained (full text unavailable n = 4; no response from the corresponding author after a single reminder n = 2). The remaining 106 reports underwent full-text eligibility assessment, of which 100 were excluded with reasons. Six studies satisfied all inclusion criteria and were included in the qualitative synthesis [19,20,21,22,23,24]. The PRISMA 2020 flow diagram is presented in Figure 1.

### 3.2. Characteristics of Included Studies

The six included studies contributed analysable HRQoL data on 1271 patients and were conducted in Europe (n = 4: the Netherlands, Germany, Croatia and France), Asia (n = 1: South Korea) and the Middle East (n = 1: Saudi Arabia). Three were prospective cohort studies [19,23,24], two were cross-sectional analytical studies [21,22] and one was a randomised controlled trial [20]. Analysed sample sizes ranged from 47 to 555. Age was reported inconsistently: three studies reported a mean (64.2 to 67.1 years), one reported a median (68 years), and two reported age only in categorical bands. The proportion of male participants ranged from 55% to 90%. Follow-up ranged from the end of the post-operative cardiac rehabilitation period to 10 years. Instruments differed across studies: the SF-36 was used in three [19,22,24], the SF-36 together with the SF-12 in one [23], the SF-12v2 in one [21] and the disease-specific MacNew questionnaire in one [20]. Because the SF-12 and SF-12v2 yield component summaries only, domain-level data were available from four studies. Study characteristics are summarised in Table 3.

### 3.3. Clinical and Methodological Heterogeneity

Heterogeneity across the six studies was substantial and is summarised in Table 4 across the five pre-specified domains. It is described here in detail because it constrains every subsequent statement in this review.

Design heterogeneity. The included studies span three designs with different inferential capacity: one randomised controlled trial, three prospective cohorts with pre-operative baseline measurement, and two cross-sectional studies without baseline measurement. Only the randomised trial can support a causal interpretation, and the two cross-sectional studies cannot establish temporal ordering between candidate factor and outcome.

Population and case-mix heterogeneity. Analysed samples ranged from 47 to 555 patients, a greater-than-tenfold difference, so that the two largest cohorts [19,24] contribute most of the information in this review while the smallest [23] is powered only to detect large effects. Eligibility differed materially: one cohort restricted enrolment to elective patients aged 30–75 years with ejection fraction above 20% [23]; the trial required stable coronary disease, an exercise-induced angina threshold of at least 50 W and ejection fraction of at least 35% [20]; and another cohort included urgent and emergency as well as elective surgery [19]. The proportion of male participants ranged from 55% to 90%.

Instrument heterogeneity. Four distinct instruments were used (SF-36, SF-12, SF-12v2 and MacNew). These differ in content, scaling and interpretation: the SF-36 yields eight domain scores and two component summaries; the SF-12 and SF-12v2 yield component summaries only; and the MacNew is a disease-specific instrument scored from 1 to 7 with a minimal clinically important difference of 0.5 points. Scores are not interchangeable between these instruments, and a factor identified on one cannot be assumed to behave identically on another.

Timing heterogeneity. The timing of outcome assessment ranged from the end of a three-week post-operative rehabilitation programme [20] to annual assessment over ten years [24]. This matters because the recovery trajectory is not monotonic: in the longest cohort the physical component improved to five years and then declined significantly between five and ten years, while the mental component remained improved throughout [24]. A factor measured against a three-week outcome and the same factor measured against a ten-year outcome are not estimating the same quantity, and apparent disagreement between studies may reflect timing rather than true inconsistency.

Analytical heterogeneity. Factor definition and modelling differed across every study: binary logistic regression on a dichotomised recovery/non-recovery outcome [19]; multivariable logistic regression on dichotomised ten-year scores [24]; hierarchical multiple linear regression on continuous component summaries [21]; stepwise forward linear regression on individual domain scores [22]; logistic regression on a dichotomised quality-of-life band [23]; and repeated-measures analysis of variance of between-group change [20]. Effect measures are correspondingly incommensurable—odds ratios for non-recovery in two studies, beta coefficients on different scales in three, and mean between-group differences in one. Comorbidity definitions also varied, with heart failure captured as NYHA class in one study and as a clinical diagnosis of congestive heart failure in another.

Taken together, this degree of heterogeneity is why meta-analysis was not appropriate, and it imposes a real limit on how far results can be combined even narratively. It also means that the absence of a factor from a given study frequently reflects that the factor was not measured, or was modelled differently, rather than that it was measured and found unimportant.

### 3.4. Risk of Bias Within Studies

Risk of bias was assessed across the five included observational studies using the Newcastle–Ottawa Scale, and the single randomised controlled trial was assessed using the Cochrane RoB 2 tool. Results are presented in Table 5. Two prospective cohort studies were rated at low risk of bias (Rijnhart-de Jong 8/9 and Perrotti 8/9 stars) [19,24]. The third prospective cohort was rated moderate (Pačarić 7/9 stars), principally because of limited comparability adjustment and a small single-centre sample [23]. Both cross-sectional studies were rated moderate; the Kim study scored 7/10 on the cross-sectional adaptation [21], and the Alzahrani study scored 5/10 [22], the principal limitations in the latter being a single-centre setting and a low participation rate (84 of 275 eligible patients; 31%), introducing potential responder bias. The randomised controlled trial [20] was judged low-risk across four of five RoB 2 domains, with some concerns in the missing outcome data domain owing to withdrawal after randomisation of 27 of 115 participants allocated to the intervention arm.

Response and completion rates were material to the outcome domain in three studies and are stated here for transparency: 555 of 1206 eligible isolated-CABG patients (46%) completed the SF-36 at both time points in the Rijnhart-de Jong cohort [19]; 171 of 230 randomised participants (74%) completed all four assessments in the Steinmetz trial [20]; and 84 of 275 eligible patients (31%) were analysed in the Alzahrani study [22]. In the Rijnhart-de Jong cohort, the authors report that non-responders were, in general, at significantly higher risk than responders, which raises the possibility that the true proportion of patients failing to recover was underestimated.

### 3.5. Results of Individual Studies and Synthesis of Factors

Factors associated with HRQoL in the included studies are summarised in Table 6, organised by category and by direction of association. For each factor, Table 6 reports the number of studies that examined it, the number that reported an independent association, and the type of evidence supporting it. The findings of each study are described below.

#### 3.5.1. Sociodemographic Factors

Female sex was examined in four studies [19,22,23,24] and was independently associated with poorer physical HRQoL in one [19]. In the Rijnhart-de Jong cohort, female sex was an independent risk factor for non-recovery of the SF-36 physical domain at one year; in the whole cardiac surgery cohort, the odds ratio for recovery was 0.492 (95% CI 0.307–0.789, *p* = 0.003), and the authors state that the risk factors identified in the isolated-CABG subgroup were identical. Female sex did not enter the final multivariable models of the remaining three studies [22,23,24]. Two of these were small (n = 84 and n = 47) and are likely to have been underpowered, but the third was a 272-patient cohort with a ten-year follow-up whose model retained age, diabetes and dyspnoea rather than sex [24]. We therefore describe female sex as supported by a single study rather than as a consistent predictor.

Age was examined in all six studies and was independently associated with HRQoL in one [24], in which younger age predicted a better physical component summary score at ten years (*p* = 0.044). The largest cohort explicitly did not confirm age as an independent risk factor, which its authors attributed to the inclusion of non-elective patients and to modelling age as a continuous rather than a categorical variable [19]; three further studies found no independent age effect [21,22,23].

Marital and employment status were associated with HRQoL in two cross-sectional studies. In the Korean study, the presence of a spouse was independently associated with a higher SF-12v2 physical component summary score (B = 3.82, *p* = 0.017 in the final model) [21]. In the Saudi study, widowhood independently predicted lower emotional well-being (β = −60.2, 95% CI −90.5 to −6.01, *p* = 0.030) and unemployment independently predicted lower general health perception (β = −27.3, 95% CI −43.0 to −11.5, *p* = 0.001) [22]. Because both designs were cross-sectional, these are associations rather than prognostic factors, and the widowhood estimate rests on only two widowed patients and is correspondingly imprecise.

#### 3.5.2. Clinical and Comorbidity Factors

Diabetes mellitus was examined in five studies [19,20,22,23,24] and was independently associated with poorer physical HRQoL in two [19,24]. In the Rijnhart-de Jong cohort, diabetes was an independent risk factor for non-recovery of the physical domain at one year (odds ratio for recovery 0.437, 95% CI 0.265–0.720, *p* = 0.001) [19]. In the Perrotti cohort, diabetes was associated with worse physical component summary scores at ten years (*p* = 0.008) and was the only factor associated with impaired mental component summary scores at ten years (*p* = 0.007) [24]. Diabetes was present in 69% of the Saudi cohort and 34% of the Croatian cohort but did not enter the final model of either [22,23]. Diabetes is thus the most frequently supported comorbidity in this review, with two of five studies reporting an independent association.

Heart failure and dyspnoea were associated with poorer HRQoL in two studies, although operationalised differently. Dyspnoea graded by NYHA class was associated with worse physical component summary scores at ten years (*p* = 0.011) [24], and a clinical diagnosis of congestive heart failure was independently associated with lower scores for role limitations due to physical problems (β = −36.5, *p* = 0.021), vitality (β = −27.7, *p* = 0.001), general health (β = −26.7, *p* < 0.001) and emotional well-being (β = −13.5, *p* = 0.011) [22]. Peripheral vascular disease was independently associated with lower general health perception in the same study (β = −21.0, *p* = 0.043) [22].

Lower pre-operative HRQoL was associated with the outcome in the one study that modelled it as a continuous baseline covariate [19]: each one-point increase in the baseline SF-36 physical domain score reduced the odds of recovery (odds ratio 0.954, 95% CI 0.942–0.965, *p* < 0.001), which the authors express as an approximately 5% increase in the odds of non-recovery per baseline point. This is in part a mathematical consequence of defining the outcome as improvement from baseline and should not be read as evidence that better pre-operative health is harmful. Two of the six studies had no pre-operative assessment at all [21,22].

Chronic obstructive pulmonary disease was recorded as a baseline variable in one cohort but did not reach significance in its multivariable models [24], and is therefore reported here as examined and not confirmed. Hypertension and dyslipidaemia, despite being highly prevalent, were not independently associated with HRQoL in any study that examined them [22,23].

#### 3.5.3. Psychosocial Factors

Psychosocial factors were examined in only two studies, both cross-sectional, and the evidence is correspondingly limited.

In the Korean study, social support measured with the Interpersonal Support Evaluation List-12 was the strongest independent correlate of the SF-12v2 mental component summary (B = 0.71, *p* = 0.002), and cardiac self-efficacy measured with the Cardiac Self-Efficacy Scale was independently associated with both the mental (B = 0.38, *p* = 0.001) and physical (B = 0.25, *p* = 0.005) component summaries [21]. Symptom experience, a composite of the frequency and severity of ten common post-CABG symptoms, was independently and inversely associated with the physical component summary (B = −0.73, *p* = 0.007) [21].

Depressive symptoms were not established as an independent factor by any included study. The only study to measure depression as a distinct construct used the Hospital Anxiety and Depression Scale and reported that it was not significant on multivariable analysis [24]. In the Korean study, depression was one of ten symptoms contributing to a composite symptom score and was not modelled separately [21]. Depressive symptoms are therefore not listed among the supported factors in Table 6. The construct remains plausible and is well supported in the wider cardiac surgery literature, but it is not established by the studies included here.

Perceived health status was a strong correlate of both component summaries in the Korean study [21], but it overlaps conceptually with HRQoL itself and we do not treat it as an explanatory factor.

#### 3.5.4. Surgical Factors

Surgical and intra-operative factors were examined in a single study, which found no significant association between left ventricular ejection fraction, the number of grafts, cardiopulmonary bypass time or aortic cross-clamp time and any of the eight SF-36 subscales [22]. Conduit strategy—arterial versus venous grafting—was not examined as a factor in any included study, a notable gap given the established differences in graft patency. On-pump versus off-pump status was likewise not examined; the ROOBY trial reported quality-of-life and neuropsychological outcomes by pump strategy but falls outside our inclusion window [25]. The evidence on surgical factors is therefore limited to one small cross-sectional study reporting null associations, and should not be read as establishing that surgical technique does not influence HRQoL.

#### 3.5.5. Post-Operative and Recovery Factors

Post-operative complications were independently associated with poorer HRQoL in two studies. In the Rijnhart-de Jong cohort, any post-operative infection—defined as any registered infection irrespective of site or severity, rather than deep sternal wound infection specifically—was an independent risk factor for non-recovery of the physical domain (odds ratio for recovery 0.240, 95% CI 0.109–0.525, *p* < 0.001) [19]. In the Saudi cohort, the development of post-operative complications, most commonly bleeding, independently predicted lower physical functioning (β = −26.3, *p* = 0.028) and lower vitality (β = −24.0, *p* = 0.043) [22].

Repeat percutaneous coronary intervention within one year of surgery was an independent risk factor for non-recovery in one study (odds ratio for recovery 0.113, 95% CI 0.036–0.349, *p* < 0.001) [19].

Exercise-based prehabilitation is the only factor in this review supported by randomised evidence. In the Steinmetz trial, two weeks of supervised aerobic exercise before surgery produced significantly greater improvement than usual care in pre-operative functional capacity (six-minute walk distance 443.0 ± 80.1 m to 493.5 ± 75.5 m in the intervention group versus 445.6 ± 105.6 m to 459.8 ± 110.1 m in controls, *p* = 0.003) and in all MacNew quality-of-life domains during the pre-operative period (*p* < 0.001 for interaction) [20]. Two qualifications are essential. First, the between-group improvement in quality of life was 0.3 to 0.4 points, below the 0.5-point minimal clinically important difference for the MacNew instrument. Second, no significant intervention effect on quality of life was observed in the post-operative period. The trial therefore demonstrates a short-lived pre-operative benefit of uncertain clinical magnitude rather than a durable post-operative quality-of-life gain.

Cardiac rehabilitation could not be evaluated as a factor by this review. All patients received post-operative cardiac rehabilitation in three included studies: in the randomised trial both arms underwent a three-week programme [20]; in the Croatian cohort rehabilitation is universal, inpatient and state-funded [23]; and in the French cohort all patients were systematically referred to the same rehabilitation centre, which its authors list as an unmeasured confounder [24]. No included study compared patients who received rehabilitation with those who did not. The improvements observed before and after rehabilitation in the Croatian cohort are within-patient changes over time in a single arm and cannot be attributed to the programme. Structured cardiac rehabilitation is therefore classified in Table 6 as not evaluable from the included evidence, although its benefits are well established elsewhere in the cardiac surgery literature [26,27].

### 3.6. Domain-Specific Findings

Domain-level data were available from the three studies using the full SF-36 [19,22,24] and from the SF-36 administered alongside the SF-12 in a fourth [23]. The two studies using 12-item instruments report component summaries only and contribute no domain-level data [20,21].

Across the studies reporting domain scores, role limitations due to physical problems was consistently the lowest-scoring domain after surgery: it was the lowest of the eight domains after rehabilitation in the Croatian cohort (mean 48.5, 95% CI 41.0–55.9) [23] and the lowest in the Saudi cohort (mean 57.4 ± 44.7) [22]. Bodily pain was among the highest-scoring domains in both (63.8, 95% CI 56.9–70.6 [23]; 75.1 ± 29.9 [22]), consistent with relief of angina being the most immediate benefit of revascularisation, although neither study assessed pain early enough to establish how quickly this occurs.

In the Croatian cohort, mental health, vitality and social functioning improved significantly only between the pre-operative assessment and the assessment after rehabilitation, with no significant change between the pre-operative and one-month assessments, and all eight domain means remained below general Croatian population norms one year after surgery [23]. In the French cohort, the two component summaries diverged over the longer term: the physical component improved to five years and then declined significantly between five and ten years while remaining above baseline, whereas the mental component improved and remained improved at ten years [24].

These observations are descriptive, drawn from four studies using two related instruments in four countries, and are offered as a pattern worth testing rather than as an established recovery trajectory.

### 3.7. Certainty of Evidence

Applying the adapted GRADE framework described in Section 2.11, certainty was moderate for a single factor—diabetes mellitus—reflecting a concordant direction of association in two independent prospective cohorts, both at low risk of bias, with rating down by one level for imprecision and the small number of contributing studies.

Certainty was low for female sex, older age, lower pre-operative HRQoL, repeat percutaneous coronary intervention, heart failure or dyspnoea, post-operative complications, and exercise-based prehabilitation. Each was supported by one or two studies with imprecise estimates or, in the case of prehabilitation, by a single trial whose effect fell below the minimal clinically important difference and did not persist post-operatively.

Certainty was very low for all factors derived solely from cross-sectional analyses—social support, cardiac self-efficacy, marital status, unemployment, symptom burden and peripheral vascular disease—reflecting indirectness arising from the absence of temporal ordering, together with imprecision and single-study support. Certainty was very low for surgical and intra-operative factors, where a single small cross-sectional study reported null associations.

No factor reached high certainty, and we did not rate up for magnitude of effect or exposure–response gradient for any factor. The inconsistency domain is poorly informed throughout, because no factor was examined by more than five studies and most were examined by only one or two. We have therefore reported, for every factor in Table 6, both the number of studies that examined it and the number that reported an independent association.

## 4. Discussion

### 4.1. Principal Findings

This systematic review synthesised evidence from six contemporary studies—three prospective cohorts, two cross-sectional analytical studies and one randomised controlled trial—examining factors associated with HRQoL after isolated CABG. Three findings emerge, and all three are qualified by the small and heterogeneous evidence base.

First, the most frequently supported factors were diabetes mellitus, independently associated with poorer physical HRQoL in two of the five studies that examined it, and post-operative complications including any post-operative infection, independently associated with poorer outcome in two of the three studies that examined them. Both cohorts supporting the diabetes finding were at low risk of bias. No other factor was supported by more than one study.

Second, the psychosocial factors most often proposed as intervention targets—social support, self-efficacy, marital status and employment—were each examined in only one or two studies, and in every case by cross-sectional designs that cannot establish whether the factor preceded the outcome. These are associations of interest, not demonstrated prognostic factors. Depressive symptoms, frequently cited in this literature, were not confirmed as an independent factor in the only included study that modelled them separately.

Third, only one factor rests on randomised evidence. Two weeks of exercise-based prehabilitation improved pre-operative functional capacity and quality of life relative to usual care, but the quality-of-life difference did not reach the minimal clinically important difference for the instrument used and no significant effect persisted into the post-operative period. No included study was able to evaluate post-operative cardiac rehabilitation, because every patient received it wherever it was delivered.

### 4.2. Comparison with Prior Reviews

Two previous syntheses provide context for these findings. Schmidt-RioValle and colleagues reviewed 16 studies and meta-analysed three to describe global HRQoL change after CABG [8]. Their work demonstrated that CABG produces clinically meaningful HRQoL improvement on average but did not focus on patient-level predictors of that improvement. Sanders and colleagues systematically reviewed 41 studies of HRQoL predictors across the broader spectrum of adult cardiac surgery, identifying 103 independent predictors [9]. Approximately half of those studies were conducted in CABG populations, but the inclusion of valve and combined cardiac surgery introduced clinical heterogeneity. The present review extends these prior syntheses by focusing exclusively on isolated CABG, applying a single risk-of-bias framework consistent with PRISMA 2020 [10], and classifying each factor by the type of evidence supporting it.

Our conclusions are more conservative than those of either prior review, and the reason is methodological rather than substantive. Restricting inclusion to isolated CABG reduces the evidence base sharply, and requiring that a factor be reported as independently associated with the outcome in the specific study to which it is attributed removes several factors that recur in narrative accounts of this literature. Depression and cardiac rehabilitation are the clearest examples: both were examined in the studies included here, but the former was not confirmed on multivariable analysis and the latter was not evaluable by design. We regard the convergence with prior reviews on pre-operative HRQoL and post-operative complications as the more meaningful agreement, precisely because those factors survive this stricter attribution standard.

### 4.3. Clinical and Methodological Implications

The clinical implications of this review are modest and should be read as hypotheses for evaluation rather than as recommendations for practice.

Diabetes and post-operative complications are the two factors with the most support, and both are already targets of established peri-operative care: glycaemic optimisation and infection-prevention bundles are recommended on other grounds, and this review adds patient-reported recovery as a further reason to pursue them. Because both associations derive from observational data, it does not follow that modifying them will improve quality of life by the amount the observed associations imply.

Pre-operative assessment of functional capacity is supported by the single randomised trial in this review to the extent that prehabilitation improved pre-operative capacity and quality of life [20]. Whether that translates into better post-operative quality of life is not established by that trial, which found no post-operative effect. Inspiratory muscle training and broader cardiac rehabilitation programmes improve respiratory muscle strength and functional capacity in adjacent systematic reviews [26,27], and their integration into post-CABG pathways rests on that evidence rather than on anything demonstrated here.

Routine assessment of social support, self-efficacy and mood is inexpensive and low-risk, and the cross-sectional associations reported here make such assessment reasonable to study. We would not, on this evidence, assert that these are established intervention targets. The appropriate next step is a prospective cohort with pre-operative measurement of these constructs, followed by randomised evaluation of any factor that survives that test.

The persistence of low scores in the role-physical domain across four studies in four countries argues for follow-up assessment that looks beyond component summary scores, since the SF-12 and SF-12v2 used in two included studies cannot detect domain-level effects at all.

From a methodological perspective, several issues recur. The use of heterogeneous HRQoL instruments across studies undermines quantitative synthesis. Consensus on a small set of core instruments for cardiac surgery, perhaps anchored on the SF-36 with the disease-specific HeartQoL as a complementary measure, would substantially improve evidence synthesis [28]. Similarly, variation in factor operationalisation—for example, depression measured by HADS or embedded within a composite symptom score, and heart failure captured as NYHA class or as a clinical diagnosis—makes pooling difficult. Adoption of a core outcome and core predictor set, developed using COMET-style consensus methodology, would address these gaps [29].

A further and easily remedied problem is that primary studies rarely report the factors they examined and did not confirm. In preparing this review, distinguishing a factor that was tested and found non-significant from one that was never measured required reading full texts and supplementary tables, and in some cases remained ambiguous. Routine reporting of the full candidate variable list alongside the final model, and explicit statement of the instrument version administered, would materially improve the quality of future syntheses in this field.

### 4.4. Context-Specific and Cross-Cultural Considerations

The six included studies were conducted in the Netherlands, Germany, South Korea, Saudi Arabia, Croatia and France—settings that differ in health system financing, rehabilitation provision, family structure, and cultural and religious context. Factors that recur across such heterogeneous settings may be closer to fundamental determinants of recovery than factors identified within a single health system. Diabetes, post-operative complications and the persistent weakness of the role-physical domain each appeared in more than one country and more than one health system, and have at least that claim on generality.

The included studies also contain evidence that context shapes these outcomes, and three examples are worth drawing out.

Rehabilitation provision differs structurally. In Croatia, cardiac rehabilitation after CABG is universal, inpatient, approximately three weeks in duration and fully funded by the health system [23]. In Germany, both arms of the included randomised trial proceeded to a three-week rehabilitation programme as routine care [20]. In France, all patients from the included centre were systematically referred to a single rehabilitation centre [24]. By contrast, the authors of the Saudi study identify inadequately structured cardiac rehabilitation and lifestyle-modification programmes as a limitation of their setting [22]. Rehabilitation is therefore a near-universal background condition in three European cohorts and an identified gap in another setting—which is precisely why it could not be evaluated as a factor here, and why findings from systems with universal rehabilitation may not transport to systems without it.

Social and family structure differ in ways that may modify measured associations. The spouse and social-support findings came from the Korean cohort, in which 70.9% of participants had a spouse and the authors interpret spousal presence as a proxy for day-to-day caregiving [21]. In the Saudi cohort, 88.1% of patients were married and only two were widowed, yet widowhood carried the largest coefficient of any variable in that study [22]. The Croatian authors attribute persistently low social-functioning scores partly to inadequate community support [23]. Whether social support operates with the same magnitude in settings with different norms of co-residence and family caregiving cannot be determined from these data.

Measurement itself may not be culturally invariant. The Croatian authors note explicitly that HRQoL does not improve in a linear way for all patients and that the norms against which scores are interpreted differ between countries [23], and they benchmark their results against Croatian rather than international population norms. The HeartQoL instrument was developed across 22 countries and 15 languages precisely because cross-national comparability of cardiac quality-of-life measurement cannot be assumed [28]. Differences in absolute scores between the studies in this review—for example, the markedly lower domain scores in the Croatian cohort than in the Saudi cohort—may reflect differences in response norms, translation and reference expectations as much as differences in underlying health.

These observations are hypothesis-generating. With one study per country, country, health system, study design and instrument are completely confounded, and this review cannot separate them. A properly designed test would require either individual participant data from multiple countries using a common instrument, or a purpose-built multinational cohort. We regard that as among the more interesting questions this literature has left open.

### 4.5. Strengths and Limitations

The principal strengths of this review are its restriction to isolated CABG, its dual independent screening and extraction, and its application of a single coherent risk-of-bias and certainty framework. A further strength is that every factor is reported with the number of studies that examined it, the number that confirmed it and the type of evidence supporting it, so that readers can judge directly how much evidence underpins each. Several limitations must nonetheless be acknowledged.

First, the small number of eligible primary studies (n = 6) restricts the generalisability of the synthesis and precluded meta-analysis. The reasons for this small yield merit comment: restriction to isolated CABG excluded the substantial body of work pooling CABG with valve and combined surgery; the requirement for explicit analysis of candidate factors excluded descriptive HRQoL studies; and the requirement for validated multidimensional instruments excluded studies using single-domain scales only. While these restrictions strengthen internal validity, they reduce the evidence base and may have excluded smaller but informative studies.

Second, language restriction to English, Spanish and Portuguese, though pragmatic, may have introduced language bias. Third, heterogeneity of HRQoL instruments and factor definitions precluded quantitative synthesis, and consequently no assessment of publication bias was possible.

Fourth, generalisability is limited in ways that go beyond the number of studies. Four of the six studies were conducted in European health systems, with one each from East Asia and the Middle East; no study from Africa, South Asia, Latin America, China or North America met our criteria. All six were single-centre. The combined evidence therefore describes tertiary-centre practice in six high- or upper-middle-income settings, and may not describe CABG recovery in health systems with different access to rehabilitation, different post-discharge follow-up or different population risk profiles.

Fifth, the sex distribution of the included cohorts constrains inference precisely where it matters most. Male participants comprised 55% to 90% of the included samples, and more than three-quarters in four of the six studies. Female sex emerged as a factor associated with poorer physical recovery in the one study that confirmed it, yet the subgroups on which such estimates rest are small—13 women in one cohort and 21 in another. The evidence base is least informative about the group it identifies as being at greatest risk.

Sixth, we were unable to evaluate several factors that the wider literature treats as important—depression, cardiac rehabilitation and conduit strategy among them—not because they were tested and found unimportant, but because they were not tested in an evaluable way in the studies meeting our criteria. Readers should not infer absence of effect from absence of evidence.

Seventh, our extraction relies on published reports and Supplementary Materials. Where a study reported effect estimates only for a pooled cohort and stated that subgroup risk factors were identical, we have reported the pooled estimates and labelled them as such, rather than attributing subgroup-specific values that were not published [19].

Finally, certainty of evidence under GRADE remained low to very low for all but one factor, reflecting the predominantly observational and frequently cross-sectional nature of the included evidence.

### 4.6. Implications for Future Research

Three research priorities follow from this synthesis. First, harmonised reporting of HRQoL and candidate factor sets, through adoption of a core outcome set for cardiac surgery, would enable future meta-analytic synthesis. Second, large multicentre cohort studies of isolated CABG with prospective enrolment across diverse healthcare systems are needed, particularly in low- and middle-income countries where the burden of ischaemic heart disease is rising fastest [2]. Third, randomised trials of integrated prehabilitation programmes—combining exercise, inspiratory muscle training, psychological support and structured education—should use HRQoL as a primary endpoint with both global and domain-specific outcomes pre-specified, should report whether effects exceed the minimal clinically important difference, and should stratify analyses by the baseline factors identified in this review.

Fourth, and most immediately, primary studies in this field should report the full set of candidate variables examined, not only those retained in final models, and should state the instrument and version administered. In preparing this review, two of the six included studies required full-text and Supplementary Materials reviews to establish which instrument had been used.

## 5. Conclusions

Across six studies of isolated coronary artery bypass grafting, the factors most often associated with poorer post-operative health-related quality of life were diabetes mellitus and post-operative complications, each supported by two independent studies. Female sex, older age, lower pre-operative quality of life, repeat percutaneous coronary intervention, and heart failure or dyspnoea were each supported by a single study. Psychosocial correlates—social support, cardiac self-efficacy, marital status and employment—were identified only in cross-sectional analyses and cannot be interpreted as prognostic. The single randomised trial showed that pre-operative exercise improves quality of life before surgery by an amount below the minimal clinically important difference, with no demonstrated post-operative effect.

The evidence base is small, methodologically heterogeneous and almost entirely observational, and it does not yet support confident statements about which factors determine quality of life after CABG, still less about which can be modified to improve it. What it does support is a clear research agenda: prospective, multicentre cohorts using a common instrument with pre-operative baseline measurement, reporting all candidate variables examined, followed by randomised evaluation of the psychosocial and rehabilitation factors that survive that scrutiny. Until then, pre-operative assessment of quality of life, functional capacity and social circumstances is best regarded as good clinical practice and a research priority rather than as an evidence-based intervention.

## Figures and Tables

**Table 1:** Eligibility criteria (PICOS framework).

Element	Inclusion Criteria	Exclusion Criteria
Population	Adults (≥18 years) undergoing isolated coronary artery bypass grafting (on- or off-pump; first-time or redo).	Paediatric patients; combined cardiac procedures (e.g., CABG with valve repair/replacement or aortic surgery) where outcomes for the CABG subset were not separately reported; heart transplantation.
Intervention/Exposure	Isolated CABG, with or without an adjunctive recovery intervention (e.g., prehabilitation, cardiac rehabilitation, inspiratory muscle training).	Percutaneous coronary intervention (PCI) without a CABG comparator arm; pharmacotherapy-only cohorts.
Comparator	Pre-operative baseline; usual care; alternative recovery intervention; or none (for single-arm cohorts).	—
Outcome	HRQoL measured with a validated multidimensional instrument (e.g., SF-36, SF-12, EQ-5D, NHP, MacNew, HeartQoL), with at least one analysis of a candidate factor (univariable or multivariable).	HRQoL not reported; non-validated instruments; single-domain symptom scales used in isolation; analysis of candidate factors not conducted or not reported.
Study design	Prospective or retrospective cohort, cross-sectional analytical, or randomised controlled trial. Published in English, Spanish or Portuguese; 1 January 2010 to 31 March 2025.	Case reports/series (n < 20); narrative reviews; editorials; commentaries; conference abstracts only; study protocols without results; duplicate publications of the same cohort (most complete report retained).

**Table 2:** Full electronic search strategies (final search executed 31 March 2025).

Database	Search Strategy
PubMed/MEDLINE	#1 “Coronary Artery Bypass”[MeSH] OR “coronary artery bypass”[tiab] OR CABG[tiab] OR “aortocoronary bypass”[tiab] OR “myocardial revascularization”[tiab] #2 “Quality of Life”[MeSH] OR “quality of life”[tiab] OR HRQoL[tiab] OR HRQOL[tiab] OR “health-related quality of life”[tiab] OR “health status”[tiab] OR “patient reported outcome*”[tiab] OR SF-36[tiab] OR SF-12[tiab] OR EQ-5D[tiab] OR “Nottingham Health Profile”[tiab] OR MacNew[tiab] OR HeartQoL[tiab] #3 predict*[tiab] OR determinant*[tiab] OR factor*[tiab] OR associat*[tiab] OR “risk factor*”[tiab] OR “protective factor*”[tiab] #4 #1 AND #2 AND #3 Filters: English, Spanish, Portuguese; humans; from 1 January 2010 to 31 March 2025. Records retrieved: n = 412.
Embase (Elsevier)	#1 ‘coronary artery bypass graft’/exp OR ‘coronary artery bypass’:ti,ab OR CABG:ti,ab OR ‘aortocoronary bypass’:ti,ab OR ‘myocardial revascularization’:ti,ab #2 ‘quality of life’/exp OR ‘quality of life’:ti,ab OR HRQoL:ti,ab OR HRQOL:ti,ab OR ‘health-related quality of life’:ti,ab OR ‘short form 36′:ti,ab OR ‘short form 12′:ti,ab OR ‘EQ-5D’:ti,ab OR ‘Nottingham Health Profile’:ti,ab OR MacNew:ti,ab OR HeartQoL:ti,ab #3 predict*:ti,ab OR determinant*:ti,ab OR factor*:ti,ab OR associat*:ti,ab #4 #1 AND #2 AND #3 Limits: human; English/Spanish/Portuguese; 2010–2025; Embase only (not MEDLINE-only records, to reduce duplication). Records retrieved: n = 538.
Cochrane CENTRAL	#1 MeSH descriptor: [Coronary Artery Bypass] explode all trees #2 (“coronary artery bypass” OR CABG OR “aortocoronary bypass”):ti,ab,kw #3 MeSH descriptor: [Quality of Life] explode all trees #4 (“quality of life” OR HRQoL OR SF-36 OR SF-12 OR EQ-5D):ti,ab,kw #5 (#1 OR #2) AND (#3 OR #4) Limits: trials; publication year 2010–2025. Records retrieved: n = 87.
CINAHL (EBSCO)	S1 (MH “Coronary Artery Bypass+”) OR TI “coronary artery bypass” OR AB “coronary artery bypass” OR TI CABG OR AB CABG S2 (MH “Quality of Life+”) OR TI “quality of life” OR AB “quality of life” OR TI HRQoL OR AB HRQoL OR TI “SF-36” OR AB “SF-36” S3 TI (predict* OR determinant* OR factor* OR associat*) OR AB (predict* OR determinant* OR factor* OR associat*) S4 S1 AND S2 AND S3 Limits: peer-reviewed; English/Spanish/Portuguese; 2010–2025. Records retrieved: n = 174.

**Table 3:** Characteristics of the six included studies.

Study (Year, Country)	Design	N Analysed	Age; % Male	HRQoL Instrument; Follow-Up	Factors Examined
Rijnhart-de Jong et al. 2020 (Netherlands) [19]	Prospective cohort, single centre	555 isolated-CABG patients with paired questionnaires (of 1206 eligible; 46%). The whole cardiac surgery cohort comprised 803 responders	Median 68 years (IQR 62–74); 84% male	SF-36; pre-operative and 1 year	Demographics, comorbidities, EuroSCORE II components, operative variables, post-operative infection, PCI within 1 year
Steinmetz et al. 2020 (Germany) [20]	RCT (exercise-based prehabilitation vs usual care)	230 randomised; 203 analysed at baseline; 171 completed all assessments	67.1 ± 8.4; 90% male	MacNew Heart Disease HRQoL questionnaire; baseline, day before surgery, start and end of cardiac rehabilitation	Exercise-based prehabilitation (randomised); functional capacity (6MWT, Timed-Up-and-Go); cardiopulmonary exercise capacity
Kim et al. 2022 (South Korea) [21]	Cross-sectional analytical	110	64.2 ± 9.6; 82% male	SF-12v2; single assessment within 12 months of CABG	Presence of spouse, post-CABG duration, work status, perceived health status, symptom experience, cardiac self-efficacy, social support
Alzahrani et al. 2024 (Saudi Arabia) [22]	Cross-sectional, descriptive-correlational	84 (of 275 eligible; 31%)	Reported in bands (45% aged 50–60; 43% >60); 85% male	SF-36; single assessment ≥1 year after CABG	Sociodemographics (age band, sex, marital status, employment), comorbidities (heart failure, diabetes, hypertension, peripheral vascular disease), LVEF, number of grafts, CPB and cross-clamp time, post-operative complications
Pačarić et al. 2020 (Croatia) [23]	Prospective cohort, single centre	47	Reported in bands (49% aged >60); 55% male	SF-36 and SF-12 (SF-6Dv2 for QALYs); pre-operative, 1 month, after rehabilitation (1 year)	Age, sex, living alone, smoking, hypertension, diabetes, hyperlipidaemia
Perrotti et al. 2019 (France) [24]	Prospective cohort, long-term follow-up	272	65 ± 10; 78% male	SF-36; pre-operative, then annually to 10 years	Age, sex, marital status, education, employment, diabetes, COPD, peripheral artery disease, atrial fibrillation, anxiety and depression (HADS), angina (CCS), dyspnoea (NYHA), LVEF, surgical complications

CABG, coronary artery bypass grafting; CCS, Canadian Cardiovascular Society; COPD, chronic obstructive pulmonary disease; CPB, cardiopulmonary bypass; HADS, Hospital Anxiety and Depression Scale; IQR, interquartile range; LVEF, left ventricular ejection fraction; NYHA, New York Heart Association; PCI, percutaneous coronary intervention; QALY, quality-adjusted life-year; RCT, randomised controlled trial; 6MWT, six-minute walk test. Where a study reported both a whole-cohort and an isolated-CABG analysis, the isolated-CABG population is reported here.

**Table 4:** Clinical and methodological heterogeneity across the six included studies.

Domain	Range Observed Across Studies	Implication for Synthesis
Study design	1 randomised controlled trial; 3 prospective cohorts; 2 cross-sectional analytical studies	Only the trial supports causal inference; cross-sectional studies cannot establish temporal ordering between factor and outcome
Analysed sample size	47 to 555 patients	More than tenfold variation; the two largest cohorts dominate the synthesis, the smallest is powered only for large effects
Case-mix and eligibility	Elective only vs elective plus urgent and emergency; ejection fraction thresholds >20% or ≥35%; upper age limits of 75 or 85 years; angina threshold ≥50 W in one trial	Populations are not exchangeable; differences in eligibility may generate apparent disagreement between studies
Sex distribution	55% to 90% male	Estimates for female patients rest on small subgroups in most cohorts
HRQoL instrument	SF-36; SF-36 plus SF-12; SF-12v2; MacNew	Scores are not interchangeable; domain-level data unavailable from the two 12-item instruments
Pre-operative (baseline) measurement	Present in 4 studies; absent in 2	Change scores and prognostic interpretation are possible only where a baseline exists
Timing of outcome assessment	End of a 3-week rehabilitation programme to annual assessment over 10 years	Different estimands; the recovery trajectory is not monotonic, so timing alone can produce apparent inconsistency
Outcome metric	Dichotomised recovery/non-recovery; dichotomised score band; continuous component summary; continuous domain score; between-group change	Effect measures (odds ratios vs. beta coefficients vs. mean differences) are not poolable
Statistical model	Binary logistic; multivariable logistic; hierarchical linear; stepwise forward linear; repeated-measures ANOVA	Adjustment sets differ; stepwise selection risks optimistic inclusion
Factor definition	Heart failure as NYHA class vs clinical congestive heart failure diagnosis; depression via HADS vs embedded in a composite symptom score	The same label does not denote the same construct across studies

ANOVA, analysis of variance; HADS, Hospital Anxiety and Depression Scale; HRQoL, health-related quality of life; NYHA, New York Heart Association.

**Table 5:** Risk-of-bias assessment of included studies.

Study	Tool	Selection	Comparability	Outcome/Exposure	Overall RoB
Rijnhart-de Jong 2020 [19]	NOS (cohort)	4/4	2/2	2/3	Low (8/9)
Steinmetz 2020 [20]	RoB 2 (RCT)	Low	Low	Low (outcome); some concerns (missing data)	Low overall
Kim 2022 [21]	NOS (cross-sectional)	3/5	2/2	2/3	Moderate (7/10)
Alzahrani 2024 [22]	NOS (cross-sectional)	2/5	1/2	2/3	Moderate (5/10)
Pačarić 2020 [23]	NOS (cohort)	3/4	1/2	3/3	Moderate (7/9)
Perrotti 2019 [24]	NOS (cohort)	4/4	2/2	2/3	Low (8/9)

NOS, Newcastle–Ottawa Scale; RoB 2, Cochrane Risk of Bias 2 tool; RCT, randomised controlled trial. NOS scoring: cohort studies (selection 4, comparability 2, outcome 3; maximum 9 stars); cross-sectional adaptation (selection 5, comparability 2, outcome 3; maximum 10 stars).

**Table 6:** Synthesis of factors associated with HRQoL after isolated CABG, by type of evidence and number of supporting studies.

Factor	Category	Type of Evidence	Direction of Association	Studies Examining/Reporting	Certainty (GRADE)
Diabetes mellitus	Clinical	Prognostic association (2 cohorts)	↓ physical HRQoL	5/2 [19,24]	Moderate
Post-operative complications, including any infection	Post-operative	Prognostic association (cohort); association (cross-sectional)	↓ physical HRQoL and vitality	3/2 [19,22]	Low
Female sex	Sociodemographic	Prognostic association (cohort)	↓ physical HRQoL	4/1 [19]	Low
Older age	Sociodemographic	Prognostic association (cohort)	↓ physical HRQoL at 10 years	6/1 [24]	Low
Lower pre-operative HRQoL	Clinical/baseline	Prognostic association (cohort)	↓ odds of recovery	4/1 [19]	Low
Repeat PCI within 1 year	Post-operative	Prognostic association (cohort)	↓ odds of recovery	1/1 [19]	Low
Heart failure/NYHA dyspnoea	Clinical	Prognostic association (cohort); association (cross-sectional)	↓ physical and mental HRQoL	3/2 [22,24]	Low
Peripheral vascular disease	Clinical	Association (cross-sectional)	↓ general health perception	2/1 [22]	Very low
Social support	Psychosocial	Association (cross-sectional)	↑ mental HRQoL	1/1 [21]	Very low
Cardiac self-efficacy	Psychosocial	Association (cross-sectional)	↑ physical and mental HRQoL	1/1 [21]	Very low
Presence of spouse/widowhood	Psychosocial	Association (cross-sectional)	Spouse ↑ physical HRQoL; widowhood ↓ emotional well-being	3/2 [21,22]	Very low
Unemployment	Sociodemographic	Association (cross-sectional)	↓ general health perception	3/1 [22]	Very low
Symptom burden (composite)	Clinical	Association (cross-sectional)	↓ physical HRQoL	1/1 [21]	Very low
Exercise-based prehabilitation	Pre-operative intervention	Intervention effect (randomised)	↑ pre-operative HRQoL and functional capacity; below MCID; no post-operative HRQoL effect	1/1 [20]	Low
Number of grafts, CPB time, cross-clamp time, LVEF	Surgical/intra-operative	Association (cross-sectional)	No significant association	1/0 [22]	Very low
Depressive symptoms	Psychosocial	Not established	Not significant on multivariable analysis in the only study modelling it separately [24]	2/0	Not supported
Structured cardiac rehabilitation	Post-operative intervention	Not evaluable	All patients received rehabilitation in [20,23,24]; no comparison with non-receipt available	0 comparative	Not assessable

CABG, coronary artery bypass grafting; CPB, cardiopulmonary bypass; HRQoL, health-related quality of life; LVEF, left ventricular ejection fraction; MCID, minimal clinically important difference; NYHA, New York Heart Association; PCI, percutaneous coronary intervention. “Studies examining/reporting” gives the number of included studies in which the factor was examined and, after the solidus, the number reporting an independent association. ↑ denotes association with better HRQoL and ↓ with poorer HRQoL. No factor was supported by more than two studies.

## Data Availability

All data underlying this review are extracted from publicly available published primary studies, which are cited in the reference list. The extracted data set, full search strategies and the PRISMA 2020 checklist are available from the corresponding author on reasonable request.

## Supplementary Materials

The following supporting information can be downloaded at: https://www.mdpi.com/article/10.3390/jcm15218628/s1, Table S1: PRISMA 2020 Checklist.
