# Supplementary material for: Predictors of Health-Related Quality of Life After Coronary Artery Bypass Grafting: A Systematic Review
Source: J Clin Med. 2026 Nov 5;15(21):8628. doi: 10.3390/jcm15218628 (PMC13764043; doi:10.3390/jcm15218628)
Supplement: Supplementary file 1 [file jcm-15-08628-s001.zip › jcm-4569941-supplementary.pdf]

PRISMA 2020 Checklist

Section and Topic	Item #	Checklist item	Location where item is reported
**TITLE**			
Title	1	Identify the report as a systematic review.	Title, p. 1 — the title identifies the report as "… A Systematic Review"; the article type is given as "Systematic Review".
**ABSTRACT**			
Abstract	2	See the PRISMA 2020 for Abstracts checklist.	Abstract, p. 1 (structured: Background, Methods, Results, Conclusions).
**INTRODUCTION**			
Rationale	3	Describe the rationale for the review in the context of existing knowledge.	Section 1 (Introduction), pp. 1–2.
Objectives	4	Provide an explicit statement of the objective(s) or question(s) the review addresses.	Section 1.1 (Objectives), p. 2.
**METHODS**			
Eligibility criteria	5	Specify the inclusion and exclusion criteria for the review and how studies were grouped for the syntheses.	Section 2.3 and Table 1, p. 2. Grouping of factors for the synthesis is described in Section 2.10 (p. 4) and applied in Section 3.5 (pp. 8–10) and Table 6 (p. 10).
Information sources	6	Specify all databases, registers, websites, organisations, reference lists and other sources searched or consulted to identify studies. Specify the date when each source was last searched or consulted.	Section 2.4, p. 2 — PubMed/MEDLINE, Embase (Elsevier), Cochrane CENTRAL and CINAHL (EBSCO), plus reference lists of two previous systematic reviews and forward citation searching of all included studies. All sources were last searched on 31 March 2025 (Sections 2.4–2.5; Table 2, p. 3).
Search strategy	7	Present the full search strategies for all databases, registers and websites, including any filters and limits used.	Section 2.5 and Table 2, p. 3 — full line-by-line strategies for all four databases, including every filter and limit applied and the number of records retrieved from each.
Selection process	8	Specify the methods used to decide whether a study met the inclusion criteria of the review, including how many reviewers screened each record and each report retrieved, whether they worked independently, and if applicable, details of automation tools used in the process.	Section 2.6, p. 3 — records de-duplicated and screened in Rayyan; two reviewers independently screened titles and abstracts and then full texts; disagreements resolved by discussion and, where necessary, a third reviewer.
Data collection process	9	Specify the methods used to collect data from reports, including how many reviewers collected data from each report, whether they worked independently, any processes for obtaining or confirming data from study investigators, and if applicable, details of automation tools used in the process.	Section 2.7, p. 3 — standardised extraction form piloted on two studies; two reviewers extracted data independently; discrepancies resolved by consensus. Data were taken from the full text and supplementary material of each report.
Data items	10a	List and define all outcomes for which data were sought. Specify whether all results that were compatible with each outcome domain in each study were sought (e.g. for all measures, time points, analyses), and if not, the methods used to decide which results to collect.	Section 2.9, p. 4 (primary outcome: HRQoL as a global or component score, or at the level of individual domains); eligibility for the outcome is defined in Table 1 (Outcome row), p. 2.
	10b	List and define all other variables for which data were sought (e.g. participant and intervention characteristics, funding sources). Describe any assumptions made about any missing or unclear information.	Section 2.7, p. 3; study- and population-level variables are presented in Table 3, p. 5. Where a study reported estimates only for a pooled cohort, this is stated and labelled rather than assumed (Sections 2.7, p. 3, and

Section and Topic	Item #	Checklist item	Location where item is reported
			4.5, p. 13).
Study risk of bias assessment	11	Specify the methods used to assess risk of bias in the included studies, including details of the tool(s) used, how many reviewers assessed each study and whether they worked independently, and if applicable, details of automation tools used in the process.	Section 2.8, p. 3 — Newcastle–Ottawa Scale (cohort version and cross-sectional adaptation) for observational studies and Cochrane RoB 2 for the randomised trial; assessed independently by two reviewers.
Effect measures	12	Specify for each outcome the effect measure(s) (e.g. risk ratio, mean difference) used in the synthesis or presentation of results.	Sections 2.9 (p. 4) and 3.3 (pp. 6–7); Table 4, outcome-metric and statistical-model rows (p. 7). Effect measures are reported as given by each source study (odds ratios, beta coefficients, mean between-group differences); no common effect measure was derived because no meta-analysis was performed.
Synthesis methods	13a	Describe the processes used to decide which studies were eligible for each synthesis (e.g. tabulating the study intervention characteristics and comparing against the planned groups for each synthesis (item #5)).	Section 2.10, p. 4; grouping of factors for narrative synthesis is applied in Section 3.5 (pp. 8–10) and Table 6 (p. 10).
	13b	Describe any methods required to prepare the data for presentation or synthesis, such as handling of missing summary statistics, or data conversions.	Sections 2.7 (p. 3) and 2.9 (p. 4). No data conversions, transformations or imputation were performed; scores are reported and interpreted within each instrument and are not converted between instruments.
	13c	Describe any methods used to tabulate or visually display results of individual studies and syntheses.	Section 2.10, p. 4. Results are tabulated in Tables 3–6 (pp. 5–10) and the selection process is displayed in Figure 1 (p. 5).
	13d	Describe any methods used to synthesize results and provide a rationale for the choice(s). If meta-analysis was performed, describe the model(s), method(s) to identify the presence and extent of statistical heterogeneity, and software package(s) used.	Section 2.10, p. 4 — a pre-specified threshold for meta-analysis (at least three studies reporting the same factor on a comparable scale with a comparable outcome metric) was not met for any factor; results were therefore synthesised narratively and reported in accordance with the SWiM guideline. No meta-analysis was performed, so no model, statistical heterogeneity measure or software package applies.
	13e	Describe any methods used to explore possible causes of heterogeneity among study results (e.g. subgroup analysis, meta-regression).	Section 2.10 (p. 4) and Section 3.3 with Table 4 (pp. 6–7) — heterogeneity was examined across five pre-specified domains (design, population and case-mix, instrument, timing of outcome assessment, and factor definition and modelling). Subgroup analysis and meta-regression were not applicable, as no meta-analysis was performed.
	13f	Describe any sensitivity analyses conducted to assess robustness of the synthesized results.	Not applicable — no sensitivity analyses were conducted, as no meta-analysis was performed (Section 2.10, p. 4).
Reporting bias assessment	14	Describe any methods used to assess risk of bias due to missing results in a synthesis (arising from reporting biases).	Section 2.10, p. 4 — formal assessment of small-study effects by funnel plot was not applicable because no meta-analysis was performed.
Certainty	15	Describe any methods used to assess certainty (or confidence) in the body of evidence for an	Section 2.11, p. 4 — GRADE as adapted for prognostic-

PRISMA 2020 Checklist

Section and Topic	Item #	Checklist item	Location where item is reported
assessment		outcome.	factor questions, with starting certainty, each rating-down domain (including indirectness for cross-sectional designs and phase of investigation), the rating-up domains considered, the reviewer process and the limitations of the approach all specified.
**RESULTS**			
Study selection	16a	Describe the results of the search and selection process, from the number of records identified in the search to the number of studies included in the review, ideally using a flow diagram.	Section 3.1 (p. 4) and Figure 1 (p. 5).
	16b	Cite studies that might appear to meet the inclusion criteria, but which were excluded, and explain why they were excluded.	Figure 1 (p. 5) and Section 3.1 (p. 4) give the reasons, with counts, for all 100 reports excluded at full-text assessment. Section 3.5.4 (p. 9) cites the ROOBY trial [25], which might appear eligible, and explains why it falls outside the inclusion window. Individually cited excluded reports are not listed separately.
Study characteristics	17	Cite each included study and present its characteristics.	Section 3.2 (p. 5) and Table 3 (p. 5); the six included studies are cited as references [19–24].
Risk of bias in studies	18	Present assessments of risk of bias for each included study.	Section 3.4 (pp. 7–8) and Table 5 (p. 8), with domain-level scores for every included study and the response or completion rate relevant to the outcome domain.
Results of individual studies	19	For all outcomes, present, for each study: (a) summary statistics for each group (where appropriate) and (b) an effect estimate and its precision (e.g. confidence/credible interval), ideally using structured tables or plots.	Sections 3.5.1–3.5.5 (pp. 8–10) and Section 3.6 (p. 11); Tables 3 (p. 5) and 6 (p. 10). Effect estimates are presented with 95% confidence intervals or p-values exactly as reported by each source study.
Results of syntheses	20a	For each synthesis, briefly summarise the characteristics and risk of bias among contributing studies.	Sections 3.4 (pp. 7–8), 3.5 (pp. 8–10) and 3.7 (p. 11); Tables 5 (p. 8) and 6 (p. 10). Table 6 reports, for every factor, the number of included studies that examined it and the number reporting an independent association.
	20b	Present results of all statistical syntheses conducted. If meta-analysis was done, present for each the summary estimate and its precision (e.g. confidence/credible interval) and measures of statistical heterogeneity. If comparing groups, describe the direction of the effect.	Section 3.5 (pp. 8–10) and Table 6 (p. 10), giving the direction of association and the number of supporting studies for each factor. No meta-analysis was performed, so no summary estimate or measure of statistical heterogeneity is presented.
	20c	Present results of all investigations of possible causes of heterogeneity among study results.	Section 3.3 (pp. 6–7) and Table 4 (p. 7).
	20d	Present results of all sensitivity analyses conducted to assess the robustness of the synthesized results.	Not applicable — no sensitivity analyses were conducted, as no meta-analysis was performed (Section 2.10, p. 4).
Reporting biases	21	Present assessments of risk of bias due to missing results (arising from reporting biases) for each synthesis assessed.	Not assessed. Sections 2.10 (p. 4) and 4.5 (p. 13) state that formal assessment of bias due to missing results was not possible because no meta-analysis was performed.
Certainty of evidence	22	Present assessments of certainty (or confidence) in the body of evidence for each outcome assessed.	Section 3.7 (p. 11) and the Certainty (GRADE) column of Table 6 (p. 10).

PRISMA

PRISMA 2020 Checklist

Section and Topic	Item #	Checklist item	Location where item is reported
**DISCUSSION**			
Discussion	23a	Provide a general interpretation of the results in the context of other evidence.	Sections 4.1 (p. 11), 4.2 (pp. 11–12) and 4.4 (pp. 12–13).
	23b	Discuss any limitations of the evidence included in the review.	Sections 3.3 (pp. 6–7), 3.7 (p. 11) and 4.5 (p. 13).
	23c	Discuss any limitations of the review processes used.	Section 4.5 (p. 13) — restriction to isolated CABG and its effect on yield, language restriction, inability to assess reporting bias, reliance on published reports and supplementary material, and factors that were not evaluable from the included evidence.
	23d	Discuss implications of the results for practice, policy, and future research.	Sections 4.3 (p. 12), 4.6 (p. 14) and 5 (Conclusions, p. 14).
**OTHER INFORMATION**			
Registration and protocol	24a	Provide registration information for the review, including register name and registration number, or state that the review was not registered.	Section 2.1, p. 2 — the review was not prospectively registered in a public registry.
	24b	Indicate where the review protocol can be accessed, or state that a protocol was not prepared.	Section 2.1, p. 2 — no protocol was published or publicly archived. The eligibility criteria, search strategy, outcomes and planned approach to synthesis were agreed by all authors before the search was executed.
	24c	Describe and explain any amendments to information provided at registration or in the protocol.	Not applicable — the review was not registered and no protocol was published, so there were no registered or published items to amend.
Support	25	Describe sources of financial or non-financial support for the review, and the role of the funders or sponsors in the review.	Funding statement, p. 14 — this research received no external funding.
Competing interests	26	Declare any competing interests of review authors.	Conflicts of Interest statement, p. 14 — the authors declare no conflicts of interest.
Availability of data, code and other materials	27	Report which of the following are publicly available and where they can be found: template data collection forms; data extracted from included studies; data used for all analyses; analytic code; any other materials used in the review.	Data Availability Statement, p. 14 — the extracted data set, the full search strategies and the completed PRISMA 2020 checklist are available from the corresponding author on reasonable request. The full search strategies are additionally published in Table 2 (p. 3). No analytic code was generated, as no meta-analysis was performed.

From: Page MJ, McKenzie JE, Bossuyt PM, Boutron I, Hoffmann TC, Mulrow CD, et al. The PRISMA 2020 statement: an updated guideline for reporting systematic reviews. BMJ 2021;372:n71. doi: 10.1136/bmj.n71. This work is licensed under CC BY 4.0. To view a copy of this license, visit https://creativecommons.org/licenses/by/4.0/
